# The Joles Jewish Hospital: A Short-lived Dutch Small City Hospital With an Unusual Resurrection

**DOI:** 10.5041/RMMJ.10492

**Published:** 2023-01-29

**Authors:** Jack Y. Vanderhoek, Dick van de Kamp

**Affiliations:** 1 Professor Emeritus, Department of Biochemistry and Molecular Medicine, The George Washington University School of Medicine, Washington, D.C., USA; 2 Retired, President, Christian Union for Wood and Construction Workers, Utrecht, The Netherlands

**Keywords:** Haarlem, hospital, Jewish, old age homes, the Netherlands, WW2

## Abstract

The Joles Jewish Hospital in Haarlem (a small city in the Netherlands) was established in 1930 to provide a Jewish milieu for local patients. Mozes Joles, a wealthy Jewish businessman, bequeathed his fortune to the Haarlem Jewish community to accomplish this objective, and its spiritual leader, Rabbi Simon Philip de Vries, was the driving force in successfully achieving this goal. The Joles Hospital was forcibly closed by the Nazis in 1943, and the postwar leadership of the Haarlem Jewish community decided not to reopen it. Instead, they used the Joles inheritance to build old age homes in both Haifa, Israel, and Haarlem, thus ensuring a Jewish environment for elderly care in both locales. The realization of one man’s charitable act bettered the lives of both ill and elderly individuals.

## INTRODUCTION

It is well known that nearly all private hospitals, including Jewish ones, that were founded from the 18th through the early 20th centuries were started by groups of interested wealthy donors. History re- cords a number of examples of Dutch Jewish benefactors who donated substantial funds or bequests to such institutions. For example, in The Hague, Mr Zurkann donated fl. 100,000 in 1846 for the Jewish Old Age home for Men and Women. The same institution received a gift of fl. 200,000 in 1927 from an unknown couple, and a Ms. Davison bequeathed fl. 30,000 in 1871 for the Jewish hospital.^1^

However, it was relatively rare for a private hospital to be launched by only one benefactor. This paper describes such a case, which occurred in Haarlem, the Netherlands in the 1930s, and an unusual postscript to the bequest that established this Jewish hospital.

## THE GROWING JEWISH COMMUNITY IN HAARLEM OF THE 1800s

Haarlem, a city about 20 miles west of Amsterdam, welcomed its first Jewish inhabitants at the beginning of the 17th century.^2^ Although the Haarlem Jewish community could not compare to either Amsterdam, Rotterdam, or The Hague in terms of influence and numbers of Jewish residents, its population grew steadily. However, the Jewish residents always accounted for less than 1.5% of the total Haarlem population, whereas the typical percentage of Jews in Amsterdam was around 10% (Table 1). According to the 1930 census, the number of Haarlem Jews constituted less than 2% of the Amsterdam Jewish total. At the end of the 19th century, the Haarlem community had a synagogue and sponsored a number of charitable societies, including the Saadat Joledot ve-Choliem, that provided support for pregnant women and the indigent sick.^3^

**Table 1:** Selected Jewish and Total Population Numbers in Haarlem and Amsterdam.^3^

Years	Haarlem		Amsterdam	
	Jews	Total	Jews	Total
1809	153 (0.6%)^2^	25,417	21,441 (10.1%)	212,413
1840	418 (1.4%)*	29,435	23,176 (10.4%)	223,114
1869	571 (1.1%)*	51,591	30,039 (10.7%)	281,502
1899	819 (0.9%)	92,096	59,117 (11.1%)	531,733
1930	1,139 (0.6%)	176,622	65,558 (8.5%)	768,409
1951	260 (0.16%)	164,007^4^	11,302 (1.3%)	855,000^5^

* Includes the towns of Bennebroek, Bloemendaal, Haarlemmermeer, Heemstede, Schoten, Spaarndam, and Zandvoort.

**Figure 1:**
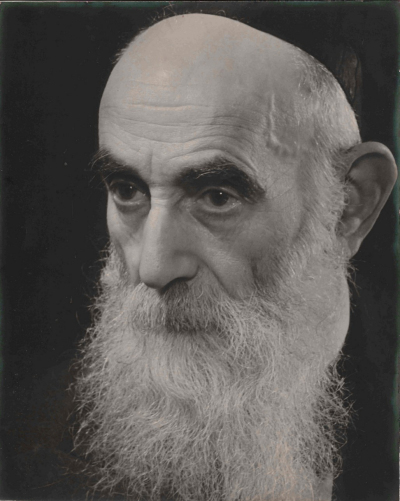
Photograph of Rabbi Simon Philip de Vries. Photo courtesy of Mrs. Tamar Walma van der Molen-de Vries.

In 1892, Simon Philip de Vries was appointed as rabbi and head teacher of the Haarlem Jewish community, a task he maintained for 48 years (Figure 1).^2^,^3^ Among his many accomplishments, two stand out. In 1905, Rabbi de Vries published a brochure titled *Ma’aneh Lezion, a defense of Zionism from a traditional Jewish point of view* in which he saw political Zionism as the only future for the Jewish people.^6^^a^ This incurred heavy criticism from the Dutch rabbinical establishment as well as most Dutch Jews, who were strongly anti-Zionist. However, this did not deter him from writing and lecturing as a passionate Orthodox Zionist, attending several international Zionist Congresses and even visiting Palestine in 1931, a 60th birthday gift from friends and students. He had a major influence on his congregants as evidenced by the fact that five of the nine members of the 1911 *kerkenraad* (governing board) of the Haarlem Jewish community were Zionists, which was highly unusual in Holland.^3^ In addition, Rabbi de Vries managed to re-awaken an interest in Jewish culture and knowledge, especially by writing many newspaper articles explaining the Jewish religion to non-Jews. These pieces were expanded into book form and resulted in his classic and popular *Jewish Rites and Symbols*, which was translated into several languages.^6^^b^

## THE JOLES FAMILY

One of the Jewish families in Haarlem in the mid-1800s was the Joles family. Born in Amsterdam in 1806, Jacob Joles had moved to Haarlem and married a local girl, Hanna de Jong in 1837.^7^ Jacob and Hanna had four children: Samuel (1843–1918), Levie/Louis (1845–1898), Mozes (1847–1927), and the youngest, Sientje, their only daughter (1851–1926). Both Samuel and Levie married and had children, but Mozes and Sientje never married and resided together in the same house at 6 Kruisstraat.^8^ Although Samuel was a member of the *kerkenraad*, it seems that Mozes was not observant but remained a communal member, a combination that was not uncommon for most Dutch Jews living in the larger cities.^3^

**Figure 2:**
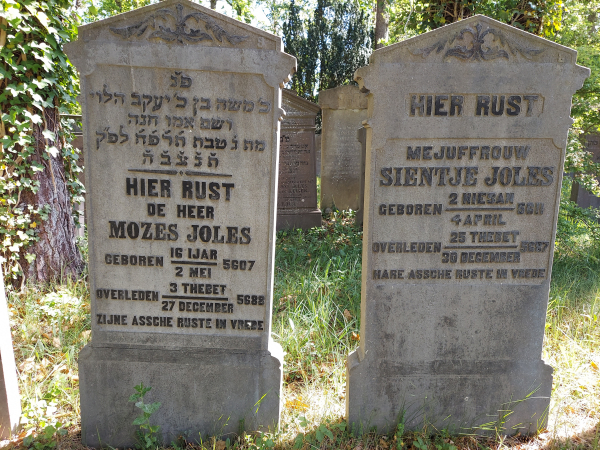
Grave Sites of Mozes and Sientje Joles in the Overveen (Netherlands) Jewish Cemetery.

The family earned their livelihood in the textile trade, but only Mozes became extremely wealthy.^9^,^10^ Whether he earned his fortune in this industry or other commercial businesses is not known. Reportedly, his assets at his death on December 27, 1927 were worth about 460,000 guilders,^11^ which would correspond to about 8.6 million euros today.^12^ The adjacent grave sites of Mozes Joles and his sister Sientje are shown in Figure 2.

## THE DUTCH JEWISH JOLES HOSPITAL BEQUEST OF MOZES JOLES

On February 18, 1927, Mozes Joles made his last will and testament and designated that his whole fortune be used to establish the *Nederlands Israelitisch Joles ziekenhuis* (Dutch Jewish Joles Hospital) in Haarlem (Robert Singer, personal communication). The deed of incorporation specified that this hospital was to provide “therapeutic treatment and compassionate nursing care at reasonable cost to all classes of patients.” Furthermore, “all patients had to be treated equally … [and] … patients had the right to choose their own physician.” [If] “sufficient space and funds were available, both healthy indigent and children could also be admitted for care.” It was also stipulated that the *kerkenraad* of the Haarlem Jewish community had overall responsibility for the hospital, including the investment of assets. The advantage of this arrangement was that the Jewish community, rather than any other group, would always have the (long-term) interests of the Jewish community truly at heart. In order to administer the Joles hospital, the *kerkenraad* of the Jewish community decided that several of its officials, i.e. the chairman (*voorzitter*), vice-chairman, and treasurer, would fulfill these same functions on the Joles board of directors. The Joles board was responsible for the hospital management (*dagelijks bestuur*). Although not specifically stated in the incorporation deed, it can be assumed that the Jewish Joles Hospital was primarily designed for Jewish patients. This was a well-established arrangement to which other confessional hospitals also adhered, so that Jews could be nursed in their own atmosphere and environment.

### The Role of Rabbi de Vries in Fulfilling the Bequest

The most suggestive explanation for Mozes Joles’s decision to found a Jewish hospital is based on a letter (dated January 30, 1928) from Rabbi de Vries to the *kerkenraad*.^13^ It should first be noted that only Amsterdam, Rotterdam, and The Hague had sufficiently large Jewish populations to support Jewish hospitals.^14^,^15^ Jewish patients who lived in other Dutch cities or villages would usually be admitted to local hospitals. Rabbi de Vries had become close to both Sientje Joles and her brother Mozes before, and especially during, Sientje’s last illness to which she succumbed on December 30, 1926. Sientje had been admitted to the Protestant Diaconessen Hospital in Haarlem just before Christmas, and Rabbi de Vries wrote that Sientje, though a non-observant Jewess, had apparently taken exception to being given a holly plant by the hospital staff, as this plant was emblematic of the Christmas holiday for Christians. This probably led to a general discussion between Mozes, Sientje, and Rabbi de Vries about the lack of a Jewish ambiance for Jewish patients in Christian hospitals. Although Rabbi de Vries pointed out that a Jewish hospital in Haarlem might not be necessary and would be quite costly, Mozes Joles indicated that the magnitude of his fortune would not be “disappointing” and he could not be dissuaded from his decision to establish a Jewish hospital in Haarlem.^16^

After the death of Mozes Joles, Rabbi de Vries took it upon himself to ensure that the Joles fortune would be used to establish an actual hospital. Despite the large monetary legacy, it seemed that this amount would not be sufficient to both build and maintain an independent Jewish hospital.^17^ Consequently, Rabbi de Vries approached an established hospital to determine whether or not they would be receptive to a collaborative arrangement such that a separate Jewish wing could be built. In the 1920s, most of the Haarlem hospitals were confessional hospitals, i.e. they were sponsored and closely associated with either a Protestant or Catholic denomination. Although the *St. Elisabeth Gasthuis of Groote Gasthuis* (St Elisabeth hospital or Great hospital) had started as a monastery hospice in 1347, it had lost its Catholic ties as a result of the Dutch Protestant reformation. In the 19th century, the municipal authorities became so involved with St Elisabeth (they had appointed the Board of Regents for centuries and occasionally became enmeshed in its daily management) that the hospital became, for all practical purposes, a municipal one, not a confessional institution.^18^ Hence, it was not surprising that Rabbi de Vries turned to St Elisabeth as a most suitable partner for implementing Joles’s vision for a Jewish hospital.

### Cooperation Between St Elisabeth Hospital and the Joles Board

In June 1928, on behalf of the Joles board of directors, Rabbi de Vries met with the Regents of St Elisabeth Hospital to discuss a possible joint arrangement.^19^^a^ He suggested that the cost of building and maintenance of a proposed independent Jewish wing^17^ at St Elisabeth would be the total responsibility of the Haarlem Jewish community. This arrangement would be advantageous to St Elisabeth for the following reasons: (1) It would free up more hospital beds for other patients (typical patient numbers at St Elisabeth were 2,121 and 2,339 in 1931 and 1932, respectively)^20^^a^ as Jewish patients would be admitted to the Joles wing; (2) It would overcome the difficulties of supplying and supervising ritual food for Jewish patients by making this the responsibility of the Joles management; and (3) Excess patients in St Elisabeth Hospital would be accommodated by available beds in the Joles wing.

The Regents were quite interested, most likely because they saw an opportunity to overcome the long-standing financial problems of St Elisabeth,^18^ and additional discussions ensued. Rabbi de Vries also represented the Joles board of directors in other negotiations with the Regents of St Elisabeth.^19^^b,c^ An agreement was finally reached in April 1929 in which the Joles hospital organization agreed to (1) purchase some properties adjacent to (and owned by) St Elisabeth in order to construct a Jewish wing connected to the main St Elisabeth hospital building, and (2) several financial arrangements with St Elisabeth to ensure the smooth management of this joint plan. Thus, the Joles board was responsible for the cost of the new building, its inventory, and the salaries of the Joles personnel (doctors, nurses, porters, kitchen staff), while St Elisabeth would be in charge of the financial management and operations (including medical services). The wing would effectively be the physical location of the new Joles Hospital. The chief medical director of the St Elisabeth *ziekenhuis* would also be in charge of the Joles wing; Joles Hospital would have use of the operating room, X-ray facility, laboratory, and two mortuary rooms of St Elisabeth. Because the Haarlem municipality had raised objections to several building plans, the start of construction was significantly delayed. Finally, on Friday, July 25, 1930, Mr A. De Lieme, the chairman of the Joles board, laid the first stone of the new Joles wing.^21^^a^ Mozes Joles had specified that the hospital had to be completed within three years after his death. Consequently, the Joles Hospital construction was finished in a record time of five months. The opening ceremony took place on December 23, 1930 and was attended by many dignitaries, including Mr De Lieme and members of the Joles board, Chief Rabbi A.S. Onderwijzer, Rabbi de Vries, the mayor of Haarlem Mr C. Maarschalk, the board chairman Mr J.H. Thyssen and members of the St Elisabeth hospital Regents, the chief medical officer Dr Kersbergen, representatives of other Haarlem hospitals, and members of the Joles family.^16^

## MANAGEMENT AND OPERATIONS OF THE JOLES HOSPITAL (WING)

**Figure 3:**
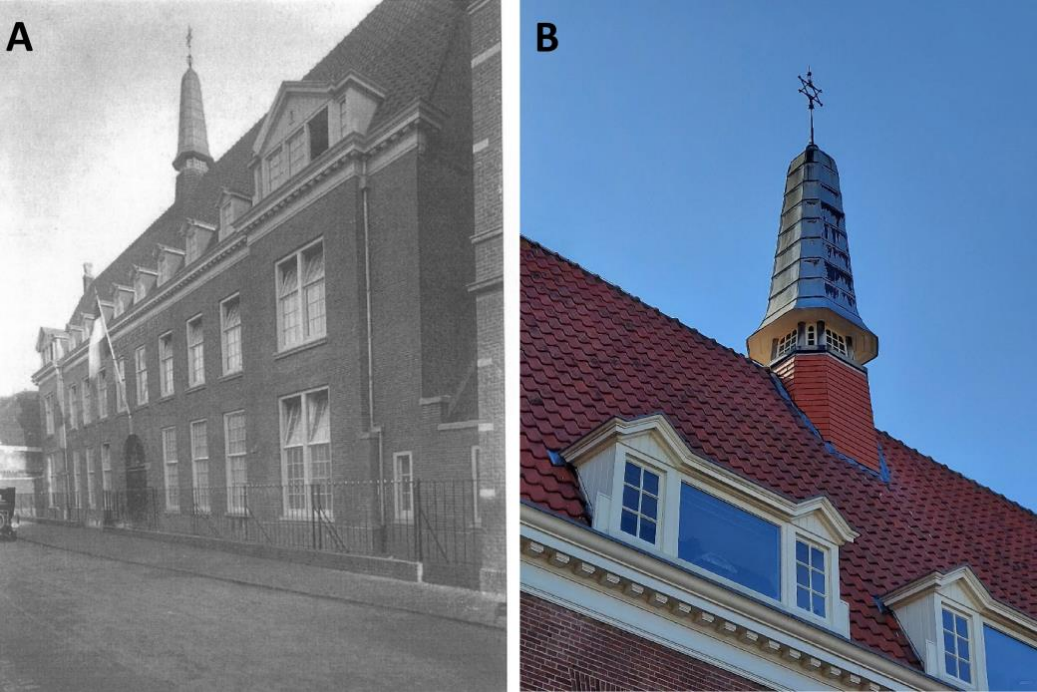
Photograph of the Joles Hospital Wing of St Elisabeth Hospital (A) and Close-up of Tower with the Jewish Star (B). Panel A reprinted from page 66 of “A Card from Haarlem,”^22^ with permission.

The Joles wing, located at 27 Groot Heiligland (Haarlem), consisted of three floors (Figure 3).^22^ An unusual characteristic of the roof was the presence of a small tower topped by a six-pointed Jewish star (Figure 3B). Interestingly, since the Joles wing was physically attached to St Elisabeth, many Haarlemmers, not knowing its history, would refer to this Jewish hospital as “St Joles.”^18^

The ground floor included three rooms for third-class patients (four beds per room and access to a terrace in the garden), the kitchen, and an isolation room. The second floor had four rooms for first- and second-class patients, each with two beds, a recreation room, a board meeting room, and a room for the rabbi to provide spiritual (and other) support for patients and their relatives. The top floor consisted primarily of rooms for nurses and storage areas.^16^ The nursing personnel tended to be mostly Jewish though not necessarily observant. The kitchen staff provided food for patients that had been prepared under rabbinical supervision. In 1937, a cardiac clinic opened, headed by Dr Hartog, a Jewish cardiac specialist.^19^^d^ Table 2 summarizes the names of the Joles Hospital administrative officers from 1928 to 1943.^19^^e–l^ Interestingly, the deed of incorporation also specified the maximum daily cost that could be charged for each class of patient, thereby ensuring the affordability of the hospital for everyone.

**Table 2:** Administrative Officers of the Nederlands Israelitisch Joles Hospital (and Their Years of Service).

Board Chairman	Chief Medical Officer	Chief Nurse
Mr A De Lieme (1928–1938)*^19^^e,g^	Dr L.C. Kersbergen (1930–1939)†^19^^f,i^	Ms M.R. van Creveld^19^^f^
Mr J.A. Rodrigues Pereira (1938–1939)^19^^h,j^	Dr P.A. Heeres (1939–1942)†^19^^i,l^	
Mr B.J. Chapon (1940–1943)*^19^^k^	Dr H.A.P. Hartog (1942–1943)*^19^^l^	

* Murdered by the Nazis.

† Also functioned as Chief Medical Officer of St Elisabeth Hospital.

As discussed above, the Haarlem Jewish community was relatively small. Since Mozes Joles had specified that the daily cost to patients had to remain low, and the number of Jewish patients averaged about four to five per day (despite the availability of 20 beds),^23^ the hospital revenue from patient fees was very limited. This financial shortfall was covered in the early 1930s by income from investment funds and rentals of farmland and several houses owned by the Joles Hospital Foundation. However, the economic crisis of the 1930s (including devaluation of the guilder) led to continued annual losses despite attempts by the Joles board to cut back. Interestingly, when asked to consider admitting Jewish patients who either lived elsewhere (e.g. Zandvoort) or were not members of the Haarlem Jewish community, the Joles board decided not to admit them. Instead they referred these patients to St Elisabeth, thereby diminishing their income.^19^^m^ It seems that people who were not members of the Haarlem Jewish community were “not entitled” to enjoy the benefits of the Joles Hospital, despite the fact that this was never stated outright in the Joles deed of incorporation. Nevertheless, if space allowed, these patients could be transferred from St Elisabeth to the Joles wing so that they too could enjoy the Jewish ritual care and ambiance. By the beginning of 1940, the Joles Hospital bequest consisted of two parts: the Joles Hospital and the Joles investment capital that also included some land holdings. The Joles Hospital carried on as well as it could, but things were about to change.

## IMPACT OF THE NAZI REGIME ON JOLES HOSPITAL

On May 10, 1940, the Germans invaded the Netherlands and conquered the country within a few weeks. One of the major goals of the German Nazi authorities was the annihilation of the Dutch Jewish community. To that end, the Nazi government isolated Jews from their Dutch compatriots by passing anti-Jewish laws; among the first was the mandatory wearing of the yellow star by Jews (April 29, 1942). Although this law did not appreciably affect the Joles Hospital, more stringent measures did. Effective April 1, 1942, the Nazis decreed that non-Jews were no longer allowed to work for Jews or Jewish institutions. As a result, several non-Jewish employees of the Joles Hospital were forced to resign.^19^^n^ To ensure that sufficient personnel were available to run the Joles Hospital, a number of nursing, kitchen, and other positions were filled by Jewish workers.^20^^b^ The next stage in the destruction of the Jewish community occurred when the Nazi regime banned the Jews from owning any property (either real estate or other assets) so that ownership of Jewish institutions, including hospitals, had to be transferred to non-Jews (May 21, 1942). As a result, the relationship between the Joles and St Elisabeth hospitals was drastically altered. Effective September 15, 1942,^19^^l^ the Joles Hospital was allowed to maintain only four patient rooms on the ground floor and two rooms on the second floor (for housing nurses van Creveld and Mok). St Elisabeth Hospital took over the rest of the second floor and the rooms on the top floor. Joles personnel (except the aforementioned two nurses) were told to live elsewhere. In November 1942, the Nazis confiscated all the Joles capital assets (worth about fl. 315,000) aside from the hospital^24^—the equivalent of 2.3 million euros in today’s currency.^12^,^24^ The Nazi occupation authorities ordered these investments (as well as all properties, e.g. bank accounts, jewelry, art collections, of all Dutch Jews and Jewish institutions) to be transferred to the Lippmann, Rosenthal & Co. bank, after which they were confiscated by the Nazi authorities. Decades after the war ended, the Dutch government reluctantly agreed to make limited restitutions for some of this looted Jewish property.^25^ The Joles foundation archives are silent as to whether such compensation was ever requested or received.

By the beginning of February 1943, all patients had been removed, and as of February 15, 1943, St Elisabeth had taken over the rest of the Joles Hospital wing. On February 25–26, 1943, all 21 Joles Jewish employees—from the chief medical officer (Dr Hartog), nurses, student-nurses, kitchen personnel, and porters to the cleaning staff—were notified that as of March 31, 1943, their jobs were terminated.^20^^c^ Joles board chairmen De Lieme and Chapon and the cardiologist Dr Hartog were all murdered by the Nazis. The *Nederlands Israelitisch Joles ziekenhuis* ceased to exist after only 13 years.

## POST WORLD WAR II: FROM JEWISH HOSPITAL TO RETIREMENT HOME

After the liberation of the Netherlands in May, 1945, and the realization that more than 80% of Dutch Jews had been murdered by the Nazis, it took time to re-establish the Dutch Jewish community. The surviving Jews were primarily those who had been hidden by a small number of compassionate and extremely brave Dutch citizens or the very few who had returned from the concentration camps or other Dutch provinces. In 1940, the Haarlem Jewish community had reached 1,460 members (presumably by the addition of some German Jewish refugees), but by 1945 less than half were left (725).^26^,^27^ If the pre-war Jewish community had difficulty supporting the Joles Hospital demographically, it now clearly became even more so. (It is interesting to note that the 1951 census indicated that the membership of the Haarlem Jewish community had further decreased to 260; see Table 1.) However, the community leadership decided that re-opening the Joles Jewish Hospital had to be considered. It is noteworthy that only one of the Jewish pre-war hospitals in Amsterdam, Rotterdam, and The Hague was restarted after the second World War—the Centrale Israelietische Ziekenverpleging (Central Jewish hospital) in Amsterdam—in contrast to the Joles Jewish Hospital, which reemerged as a Jewish old age home.

### St Elisabeth Hospital Withdraws Its Support

In 1948, the community leadership reported that: (1) the Joles funds had been confiscated by the Nazis and disappeared; (2) St Elisabeth Hospital had taken over the entire Joles wing; and (3) the St Elisabeth Regents, after several arduous discussions with the Haarlem Jewish *kerkenraad*, refused to restore the Joles wing to its original state and purpose.^28^ This refusal stood in stark contrast to the remarkable cooperation of the Regents of St Elisabeth with the Joles Jewish Hospital in the years before 1940. Several reasons can be advanced for this refusal. The excellent pre-war relationship between the Joles board and St Elisabeth was fostered by the medical director Dr Kersbergen, who was primarily an organizer and consensus builder.^29^ On the other hand, Dr Heeres, his successor, was a much more authoritarian leader who was not interested in reversing his pragmatic annexation of the Joles wing to St Elisabeth.^30^ Second, the rigid attitude of many, especially government bureaucrats and other organizations, that “everyone, not just Jews, had suffered” reflected indifference, no sense of guilt, and little understanding for the specific trauma and material loss experienced by the Jewish community.^31^

Given the small size of the postwar Haarlem Jewish community (which would result in very few Jewish patients) and the lack of financial resources, the Haarlem *kerkenraad* considered their legal options with respect to the Joles wing, since it appeared that building another Jewish hospital was neither financially nor demographically feasible. The board contacted Professor Eduard M. Meijers, the preeminent Dutch jurist, for an advisory judicial opinion as to whether or not (1) the last will and testament of Mozes Joles and the deed of incorporation of the Joles Hospital permitted the sale of the Joles Wing; and (2) if the funds from such a sale could be used for another purpose consistent with the original objectives of the Joles Hospital, such as the making of a Jewish old age home.^32^ Professor Meijers approved these options, and in 1949 the Haarlem *kerkenraad* sold the Joles wing to the Haarlem municipality (for 180,000 guilders), which in turn allowed St Elisabeth to use it as another wing of its medical complex.^11^

### A New Focus: Care for Elderly Dutch Jewish People in Israel

The Joles board decided to resurrect the Joles Hospital Foundation by launching a very different social initiative with a focus on care of the elderly instead of the original emphasis on patient care. Their decision was based on their interpretation of the phrase “healthy indigent … could also be admitted for care,” which appeared in the Joles Hospital deed of incorporation (Robert Singer, personal communication). To ensure that the recipients would be Jewish, the board established an old age home in Israel for Dutch Jews whose children had emigrated to this newly established country. The choice of Israel was not surprising for several reasons. First, the leadership of the post-war Dutch Jewish community was strongly Zionistic.^33^ Second, in view of the devastation of the Dutch Jewish community by the Nazis, a number of Jews questioned whether there was any future for Jews and Jewish institutions in Holland. Third, Rabbi de Vries had been one of the few and most pro-Zionistic rabbis in Holland, and he clearly had a major philosophical impact on his Haarlem congregants.^6^ Despite his murder by the Nazis, he undoubtedly influenced the two postwar board chairmen of the Joles Hospital Foundation, J.A. Davids and D. Heijmans,^34^ who chose Israel for the location of the old age home, thereby lending their support to Israel’s development. With the help of other charitable organizations such as the Bet Zekainim Groningen Association and the Irgun Olei Holland, the *Beth Joles* (House of Joles) Old Age home was built on Mount Carmel in Haifa’s Ahuza neighborhood. The first stone was laid on

December 20, 1953 by the mayor of Haifa, Aba Khoushy, and the first residents were received in 1955. Due to a variety of delays, the official dedication occurred on May 13, 1957. Among the dignitaries were Dr D. Heijmans, the board chairman of the Joles Hospital Foundation, the Dutch ambassador B. Backer, the vice mayor Barzilay, and the board chairman of the *Beth Joles* old age home, Dr R. Polak.^21^^b^

**Figure 4:**
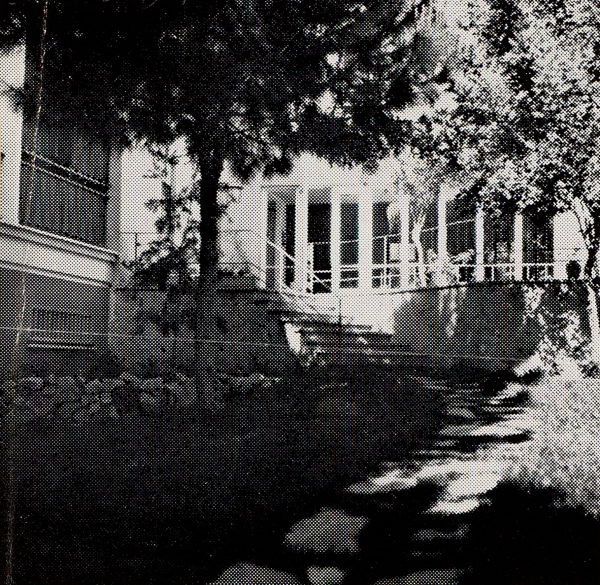
Photo of the First Beth Joles Old Age Home in Haifa, Israel. Photo courtesy of the Jewish Museum Amsterdam. Photo archive of the Nieuw Israelitisch Weekblad (New Israelite Weekly).

The home (Figure 4) initially contained 20 single and 8 double occupancy rooms, a dining room, a lounge, a small synagogue, and a nursing facility.^35^^a^ Residents were expected to pay an entry fee as well as a monthly rental fee.^35^^b^

Within 10 years, the home needed to be expanded. Between 1976 and 1979, new ground was purchased and a new eight-story building was erected; each floor contained six single rooms and two apartments for couples. Another extension was started in 1987^21^^c^ and completed in 1989. A separate *Beth Joles* Foundation, Ltd (Dutch interests) was established in Israel in 1966 (50% of which is owned by the Nederlands Israelitisch Joles Hospital Foundation) to manage the *Beth Joles* old age home and better serve the interests of the residents. The Beth Joles Foundation continues to care for the home for retired Dutch (and other) Jews in Israel.^36^

### Establishment of a Joles Retirement Home in Haarlem

**Figure 5:**
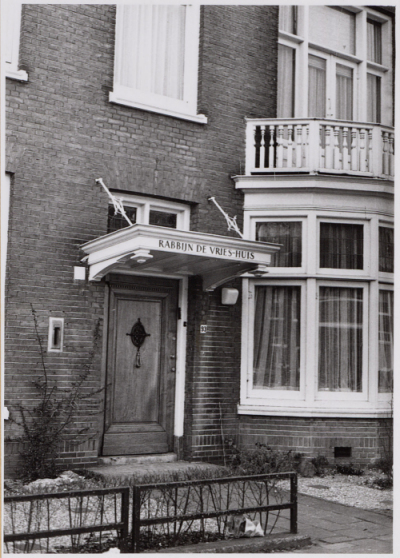
Photograph of the Rabbijn de Vrieshuis at 91–93 Verspronckweg, Haarlem, The Netherlands. Photo courtesy of the Jewish Museum Amsterdam. Photo archive of the Nieuw Israelitisch Weekblad (New Israelite Weekly).

In the mid-1960s, the board of the Joles Hospital Foundation began to focus on the needs of the rapidly increasing numbers of aging Haarlem Jews. In view of the success of the *Beth Joles* home in Israel, the board established a Jewish retirement home in the city. After considering various properties, two adjoining houses at 91 and 93 Verspronckweg were purchased and remodeled to accommodate 22 independent senior citizens. On August 30, 1970, this retirement home was inaugurated as the *Rabbijn de Vrieshuis* (Rabbi de Vries home) (Figure 5) in the presence of Mayor de Gou, Chief Rabbi E. Berlinger, and several children of Rabbi de Vries.^35^^c^,^37^ It was only fitting that the man who was the driving force behind the creation of the Joles legacy and foundation was finally honored by having his name associated with one of the projects of this organization. The home provided a strong Jewish atmosphere including a kosher kitchen and Friday evening synagogue services.^21^^d,e^ However, the *Rabbijn de Vrieshuis* was closed after 20 years due to financial problems as well as pressure from the Dutch government to centralize all Jewish old age homes. In 1991, the last residents were transferred to a separate *Rabbijn de Vries* wing of the Beth Shalom old age home in Amsterdam, which agreed to provide and maintain a Jewish atmosphere for them.^21^^f^

## SUMMARY

The legacy of Mozes Joles was originally focused on ensuring a Jewish ambiance for patients in a small Jewish hospital. This was accomplished by providing a Jewish nursing staff, ritually prepared kosher food, and the ready accessibility of the local Haarlem rabbi who provided advice and pastoral care to the patients. However, the Nazi devastation of the Dutch Jewish population in World War II led to the forced closure of the Joles Hospital. As a result of the markedly diminished financial and membership conditions of the Haarlem Jewish community, the Joles Hospital Foundation resurrected itself by shifting its focus from hospital care to delivering affordable elder care while maintaining the same emphasis on a Jewish atmosphere and milieu. This was accomplished by establishing the *Beth Joles* old age home in Haifa, Israel and subsequently the *Rabbijn de Vrieshuis* in Haarlem.

The two main figures in this history of the Joles Hospital and Foundation were Mozes Joles, its financial founder, and Rabbi de Vries, its pragmatic facilitator who ensured establishment of the Joles Hospital and whose ideological vision influenced its rebirth in both Israel and Haarlem. The Joles heritage, delivering essential medical or elderly care in a Jewish setting, is an instructive example of what a dedicated and innovative charity can accomplish.
